# Reactive Hyperplasia of the Oral Cavity: A Survey of 197 Cases in Tabriz, Northwest Iran

**DOI:** 10.5681/joddd.2010.022

**Published:** 2010-09-16

**Authors:** Amir Ala Aghbali, Sepideh Vosough Hosseini, Bahram Harasi, Maryam Janani, Seyyed Mostafa Mahmoudi

**Affiliations:** ^1^ Assistant Professor, Department of Oral Pathology, Faculty of Dentistry, Tabriz University of Medical Sciences, Tabriz Iran; ^2^ Assistant Professor, Department of Oral Pathology, Faculty of Dentistry, Tabriz University of Medical Sciences, Tabriz Iran; ^3^ Post-graduate Student, Department of Oral Pathology, Faculty of Dentistry, Tabriz University of Medical Sciences, Tabriz Iran; ^4^ Post-graduate Student, Department of Endodontics, Faculty of Dentistry, Tabriz University of Medical Sciences, Tabriz Iran

**Keywords:** Giant cell granuloma, oral lesion, pyogenic granuloma, reactive hyperplastic lesions

## Abstract

**Background and aims:**

Reactive hyperplastic lesions of the oral connective tissue are associated with injuries of soft tissue and have high prevalence rates and different involvement patterns in different parts of the world. The aim of this study was to analyze demographic data of a university department.

**Materials and methods:**

Patient records of the Department of Oral Pathology during a four-year period were reviewed for diagnosis of oral connective tissue reactive hyperplastic lesion. Data including the area involved and the type of lesion were collected and analyzed using descriptive statistical methods and t-test with SPSS 15 statistical software.

**Results:**

197 cases (mean age, 37.68±18.97; male: female ratio, 1.8:1) matched study criterion. The most common affected site was gingiva (83.9%) and the most common lesion was fibroma (45.2%). Giant cell granuloma and pyogenic granuloma were more common in the maxilla than in the mandible. Pyogenic granuloma was seen to be equally distributed in males and females.

**Conclusion:**

The results of the present study are overall consistent with the findings of previous studies.

## Introduction

Reactive hyperplasia comprises a group of fibrous connective tissue lesions that commonly occur in the oral mucosa as a result of injury.^1^ Peripheral giant cell granuloma is a reactive hyperplastic lesion containing giant cells and is caused by an unusual proliferative reaction to local irritations or trauma, though there still controversy over its nature.^2^ Another lesion is pyogenic granuloma, commonly seen in maxillary gingiva, and sometimes in pregnant women, known as granuloma gravidarum.^2^ Other common sites are mandibular gingiva, lips, tongue, and buccal mucosa, respectively.^2^ Epulis fissuratum, irritation fibroma and peripheral ossifying fibroma are the other hyperplastic lesions.^1^

It seems the prevalence of reactive hyperplastic lesions of oral connective tissue differs according to type of lesion, age, gender, and affected site. In a study carried out by Gungormus & Akgul^3^ on 27 central giant cell granuloma cases, 89% occurred before 40 years of age and 78% were seen in females and often in the mandible. The results of a study carried out by Katsikeris et al^4^ on 224 new peripheral giant cell granulomas and 959 reported instances in papers indicated that the incidence of this lesion is more common in males and in the mandible and is often detected in 40-60 year-olds. Auclair et al^5^ studied 25 central giant cell granuloma cases and indicated that this lesion occurred most in females with an average age of 21. In the study of Jalayeri et al,^6^ peripheral giant cell granuloma was more prevalent in the forth decade of life and in the mandible, and the central type was more common in females, in the second decade, and in the mandible.

Review of the literature reveals that there are controversies among the findings of previous studies, and that all reactive hyperplastic lesions have rarely been evaluated in one study. The aim of this survey was to evaluate oral connective tissue reactive hyperplastic lesions referring to a university department in the north-west of Iran during a four-year period and to compare the results with those of similar studies.

## Materials and methods

This retrospective cross-sectional study was performed on the archives of the Department of Oral Pathology at Tabriz University of Medical Sciences during the period between 2004 and 2008. Patient records were assessed to select those with the diagnosis of reactive hyperplastic lesions. Data including the type of the lesion, age, gender, and the affected site were collected using prepared forms. Descriptive statistical methods (mean, standard deviation, and percent) were applied to data, and t-test was employed to assess mean differences using SPSS 15 statistical software.

## Results

From a total of 412 records evaluated, 197 (48%) of the lesions were reactive hyperplasia. Of these, 124 (62.8%) cases were females (mean age, 39.35 ± 18.37) and 73 (37.2%) cases were males (mean age, 35.09±19.81), with a total mean age of 37.68 ± 18.97 years (minimum = 1, maximum = 82 years). There was no statistically significant difference in mean age between genders (p=0.13). The most common lesion was fibroma with 80 cases (40.7%) (Table 1). Gingiva was the most common site with 165 cases (83.9%) (Table 2). Gingival lesions in the descending order of prevalence were giant cell fibroma (39.6%), fibroma (36.5%), and pyogenic granuloma (33.3%). Of cases occurring in the gingiva and the vestibule, 79 cases were seen in the maxilla and 65 cases were observed in the mandible (Table 3).

**Table 1:** Distribution of 197 cases of reactive hyperplasia according to the type of lesion

Type of lesion	Number (%)
Fibroma	80 (40.7%)
Giant cell granuloma	64 (32.5%)
Pyogenic granuloma	42 (21.3%)
Peripheral ossifying Fibroma	2 (1%)
Epulis fissuratum	9 (4.5%)

**Table 2:** Distribution of 197 cases of reactive hyperplasia according to the site of lesion

Site of lesion	Number (%)
Gingiva	165 (83.9%)
Vestibular mucosa	16 (8.6%)
Buccal mucosa	8 (4.2%)
Tongue	5 (2.6%)
Lip	1 (0.5%)
Palate	1 (0.5%)

**Table 3:** Distribution of 144 cases in the maxillary and mandibular jaws

Site of lesion	Peripheral ossifying fibroma	Epulis fissuratum	Pyogenic granuloma	fibroma	Giant cell granuloma	Total
Maxilla	1	0	17	32	29	79
Mandible	1	1	12	26	25	65
Total	2	1	29	58	54	144

## Discussion

This survey included all types of oral connective tissue reactive hyperplastic lesions in an oral pathology department in north-west of Iran. In most areas evaluated, our results were similar to the results of others studies. However, it is remarkable that, giant cell granuloma was more common in the maxilla and pyogenic granuloma was equally distributed in males and females. Oral connective tissue reactive hyperplastic lesions were more prevalent in females than males (male-to-female ratio: 1:1.8). In a study carried out by Zarei et al,^7^ reactive hyperplastic lesions were more common in females (male-to-female ratio was 1:1.4), and the mean age of females was higher than that in males. In addition, in a study carried out by Al-khatten,^8^ the mean age of females was higher than that of males, and the most commonly affected site was gingiva (83.6%). The lesions in the order of most prevalent were as follow: giant cell granuloma (64%), fibroma (40.7%), peripheral ossifying fibroma (40%), pyogenic granuloma (21.3%), and epulis fissuratum (1%); generally, these lesions were more common in the maxilla than in the mandible.^8^ The most common site of pyogenic granuloma is gingiva, which is more prevalent in the maxilla than in the mandible.^2^ This is consistent with our results and the results of studies carried out by Saravan^7^ and Zarei.^9^

Giant cell granuloma is a more prevalent in the mandible than in the maxilla.^1^ In the current study, giant cell granuloma was a little more common in the maxilla than in the mandible, which is consistent with the results of a study carried out by Shamim et al.^10^

In the current study, fibroma was more common in the mandible than in the maxilla. In the study carried out by Shamim et al,^10^ fibroma was also more commonly found in the mandible, consistent with the results of our study.

Peripheral giant cell granuloma is often more common in females than in males.^11 - 13^ In the present study, male-to-female ratio was 1:1.4. In a study carried out by Motamedi et al^14^ the prevalence of peripheral giant cell granuloma was the same in both genders.

In a study carried out by Saravna^9^ oral pyogenic granuloma was more common in females than in males. Zhang et al^6^ found the most common gingival reactive lesion to be peripheral fibroma. In the present study, fibroma was more common in females than in males, which is consistent with a previous study.^13^

## Conclusion

Reactive hyperplastic lesions of the oral connective tissue are more common in females and the majority of the lesions occur in gingiva. Giant cell granuloma and pyogenic granuloma are more common in the maxilla whereas irritation fibroma is more commonly found in the mandible. The mean age of female patients is higher than that of males.
